# Impact of a 6-month treatment with intragastric balloon on body composition and psychopathological profile in obese individuals with metabolic syndrome

**DOI:** 10.1186/s13098-016-0197-6

**Published:** 2016-12-19

**Authors:** Erika P. Guedes, Eduardo Madeira, Thiago T. Mafort, Miguel Madeira, Rodrigo O. Moreira, Laura Maria C. Mendonça, Amélio F. Godoy-Matos, Agnaldo J. Lopes, Maria Lucia F. Farias

**Affiliations:** 1 Division of Metabology, State Institute of Diabetes and Endocrinology (IEDE), Rua Moncorvo Filho 90-Centro, Rio de Janeiro, RJ CEP 20211-340 Brazil; 2 Division of Endocrinology, Federal University of Rio de Janeiro, Rio de Janeiro, Brazil; 3 Division of Gastroenterology, State University of Rio de Janeiro, Rio de Janeiro, Brazil; 4 Division of Pulmonology, State University of Rio de Janeiro, Rio de Janeiro, Brazil; 5 Division of Rheumatology, Federal University of Rio de Janeiro, Rio de Janeiro, Brazil

**Keywords:** Obesity, Depression, Anxiety, Body composition, Intragastric balloon

## Abstract

**Background:**

The aim of this study was to investigate the effects of a 6-month treatment with intragastric balloon (IGB) on body composition and depressive/anxiety symptoms in obese individuals with metabolic syndrome (MS).

**Methods:**

Fifty patients (aged 18–50 years) with obesity and MS were selected for treatment with IGB for 6 months. Body composition was verified with dual-energy X-ray absorptiometry (DXA) at baseline and right after IGB removal. Anxiety/depressive symptoms were assessed with the Beck Depression Inventory (BDI) and the hospital anxiety and depression scale (HADS) at baseline and after 6 months of treatment.

**Results:**

In total, 39 patients completed the study. After 6 months, there were significant decreases in weight (11.7 ± 9.6 kg, p < 0.0001) and waist circumference (9.3 ± 8.2 cm, p < 0.0001). Weight loss was also demonstrated by DXA and corresponded to decreases of 3.0 ± 3.4% in body fat percentage, 7.53 ± 7.62 kg in total body fat, and 3.70 ± 4.89 kg in lean body mass (p < 0.001 for all comparisons). Depressive symptoms scores decreased by a mean of 4.57 ± 10.6 points when assessed with the BDI (p = 0.002) and 1.82 ± 5.16 points when assessed with the HADS-Depression (p = 0.0345). Anxiety symptoms scores decreased by a mean of 1.84 ± 4.04 points when determined with the HADS-anxiety (p = 0.0066). The decrease in body fat percentage was the parameter that best correlated with improvements in depressive (p = 0.008) and anxiety symptoms (p = 0.014).

**Conclusions:**

In obese individuals with MS, fat mass reduction was associated with short-term improvements in depressive and anxiety symptoms.

*Trial Registration* Registered at ClinicalTrials.gov, NCT01598233

## Background

The growing prevalence of obesity is a great concern, given the association between this disorder and several chronic diseases, including cardiovascular diseases, type 2 diabetes, hypertension, and cancer [1]. Obesity, depression, and anxiety share overlapping psychosocial and pathophysiological etiologies [2–5]. Individuals with major depressive or anxiety disorders present dysregulation of the hypothalamic–pituitary–adrenal axis with increased levels of stress-related hormones and other mediators, favoring food intake and body fat accumulation [2, 3, 6].

Several studies have found controversial results while evaluating the association of overweight and obesity with mental disorders using body mass index (BMI) [7–12]. Zhao et al. have found that BMI is an independent predictor of mental disorders, with a higher BMI showing a stronger association with depression [11]. In contrast with these findings, Papelbaum et al. found no association between BMI and depression or anxiety in a sample of 212 women seeking treatment for obesity [9]. On the other hand, measures of body composition have been associated with psychiatric symptoms [13–15]. Cugini et al. analyzed the association between anxiety and depression with body composition, assessed with bioelectrical impedance. These authors showed that anxiety and depression were influenced by relative reductions in lean mass and increase in fat mass in obese patients [13]. In a recent cross-sectional study, our group has shown that the percentage of total body fat—but not central fat, BMI, or waist circumference (WC)—was associated with an increased severity of anxiety and depressive symptoms in obese individuals with metabolic syndrome (MS) [15].

Analyses of the impact of weight loss treatment on mental health have demonstrated improvements in psychopathological parameters [16–24]. In a meta-analysis of 31 studies, Fabricatore et al. showed that most weight loss approaches had favorable effects on mood [16]. A recent systematic review analyzed eight studies directly evaluating the association between the amount of weight loss after behavioral and/or dietary interventions and the decrease in depressive symptoms. The results showed that only three out of the eight studies reported a significant positive relationship between weight loss and the degree of improvement in depressive symptoms [22]. A recent study reported no differences in depression severity between depressed and non-depressed morbidly obese women after weight loss, reflected by decreases in body fat (analyzed by bioelectrical impedance) and BMI, following treatment with IGB for 6 months. However, the degree of weight loss in the depressed group after treatment was found to have improved the depression status [24].

Based on these considerations, the aim of this study was to evaluate the association between weight loss and changes in body composition evaluated by dual-energy X-ray absorptiometry (DXA) with changes in depressive and anxiety symptoms in obese patients treated with IGB for 6 months.

## Methods

### Participants

This study comprised a consecutive sample of 50 patients who sought treatment for obesity and MS and were willing to lose weight. The participants were included after fulfilling the eligibility criteria to participate in the study and signing a written informed consent form. The protocol was approved by the Ethics Committee of the State Institute of Diabetes and Endocrinology of Rio de Janeiro, where the patients were recruited. The study was registered at ClinicalTrials.gov (NCT01598233).

The criteria for inclusion in the study comprised age between 18 and 50 years, obesity (BMI ≥ 30 kg/m^2^) and the occurrence of MS diagnosed according to the International Diabetes Federation (IDF) criteria [25, 26]. The exclusion criteria were type 1 or 2 diabetes mellitus, pregnancy or desire to become pregnant within 6 months from the enrollment, alcoholism, advanced liver disease, end-stage renal disease, current or prior coronary artery disease (defined as prior myocardial infarction, stable or unstable angina, or coronary revascularization), current or prior cerebrovascular disease (defined as prior ischemic stroke, transient ischemic attack, or carotid revascularization), history of a psychiatric disorder, current use of antidepressants or other psychiatric medications, use of antiobesity medications, and weight loss treatment in the previous 6 months [15].

### Study procedures

At the baseline evaluation (week 0), a silicone IGB (Silimed Silicone, Instrumental Médico Cirúrgico Hospital Ltda, Rio de Janeiro, RJ, Brazil) was implanted by upper gastrointestinal endoscopy under deep sedation. Under endoscopic visualization, the IGB was placed in the stomach and filled with 650 mL of normal saline solution (0.9% NaCl) and 20 mL of methylene blue solution. According to local regulations and our institutional ethics committee, all patients remained in the hospital for up to 24 h after the procedure. The patients were followed up for 6 months when the IGB was then removed via endoscopy.

### Anthropometric measures

The visits occurred at weeks 0 (baseline), 8, 16, and 24. During each visit, the following anthropometric data were recorded: body weight (kg), height (m), BMI (weight divided by the squared height), and WC (cm; determined at the midpoint between the lowest rib and the iliac crest).

### Evaluation of body composition parameters

Body composition was evaluated at weeks 0 and 24 by DXA with the densitometer Prodigy (GE Healthcare, Inc., Madison, WI, USA), and included the following analyses: body fat content (%), fat distribution, and lean mass (g).

### Assessment of anxiety and depressive symptoms

Anxiety and depressive symptoms were assessed with the hospital anxiety and depression scale (HADS), a self-report instrument to assess anxiety and depressive symptoms during the previous week. The items exclude somatic symptoms, avoiding overlap between somatic illness and mood disorders. It comprises seven statements relevant to anxiety or depression (HADS-anxiety and HADS-depression), in which each response consists of a four-point rating scale, with a higher score indicating a worse condition [27, 28]. The Beck Depression Inventory (BDI) was also used to measure the severity of the depressive symptoms. The instrument comprises 21 questions, each one with four optional answers. The total score is the sum of the scores obtained in each individual item [29]. Both questionnaires were applied at all time points (weeks 0, 8, 16, and 24).

### Statistical analysis

The statistical analysis was performed with GraphPad InStat 3.00 for Windows 95 (GraphPad Software, San Diego, CA, USA). The mean absolute change in weight, BMI, waist, total fat (in grams and percentage) and lean mass (in grams), as well as BDI, HADS-depression, and HADS-anxiety scores were analyzed from baseline to week 24 using Wilcoxon matched pairs test. Spearman’s correlation coefficient was used to determine the correlations between improvements in weight and body composition parameters and the decrease in psychiatric symptoms. The level of statistical significance was set at 5% (p ≤ 0.05).

## Results

Overall, 50 patients were included in the study. The cohort had a mean age of 34.6 ± 7.1 years and a mean BMI of 40.1 ± 6.3 kg/m^2^. The BMI was above 40 kg/m^2^ in 23 patients (46%) and below this level in 27 individuals (54%). During the 6-month follow-up period, the patients had a mean weight loss of 11.7 ± 9.6 kg (p < 0.0001), a decrease in BMI of 4.4 ± 3.5 kg/m^2^ (p < 0.0001), and a reduction in WC of 9.3 ± 8.2 cm (p < 0.0001). In total, 29 (74.35%) patients lost more than 5% of their initial weight, while 16 (41.02%) lost more than 10%, and 11 (28.20%) lost more than 15%. Variations in body composition demonstrated by DXA included a total body fat decrease of 7.53 ± 7.62 kg (p < 0.0001), corresponding to 3.0 ± 3.4% (p < 0.001), and a total lean mass decrease of 3.70 ± 4.89 kg (p < 0.001). The complete anthropometric and DXA data results have been previously published [30].

**Fig. 1:**
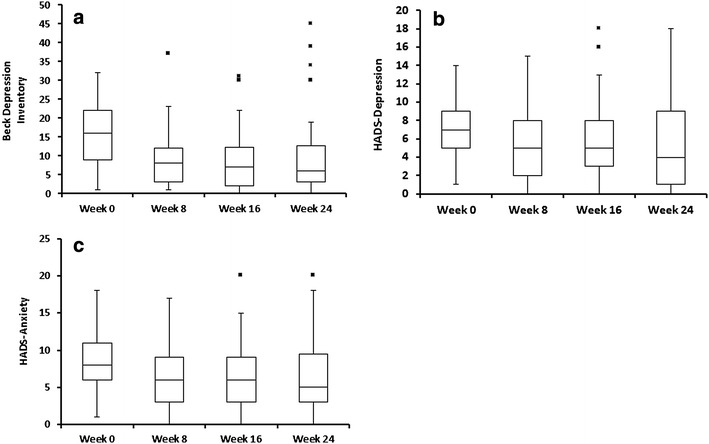
Effect of 6-month treatment with IGB in BDI (a*), HADS-D (b**) and HADS-A scores (c***). *IGB* intragastric balloon; *BDI* Beck Depression Inventory; *HADS* hospital anxiety and depression scale; *HADS-A* hospital anxiety and depression scale-anxiety; *HADS-D* hospital anxiety and depression scale-depression; * p = 0.002; ** p = 0.0345; *** p = 0.0066

Patients who completed the study also displayed significant improvements in psychiatric symptoms assessed by both psychiatric scales (Table 1; Fig. 1). Symptoms of depression decreased by an average of 4.57 ± 10.6 points in the BDI (p = 0.002; Fig. 1a) and 1.82 ± 5.16 points in the HADS-depression (p = 0.0345; Fig. 1b). Symptoms of anxiety decreased by an average of 1.84 ± 4.04 points in the HADS-anxiety (p = 0.0066; Fig. 1c).

**Table 1:** Patients’ characteristics at baseline and 6 months after treatment with intragastric balloon (IGB)

	Baseline (n = 50)	6 months (n = 39)	*p* value
BDI	16 (1–32)	6 (0–45)	0.0019
HADS-depression	7 (1–14)	4 (0–18)	0.0345
HADS-anxiety	8 (1–18)	5 (0–20)	0.0066

These results refer only to patients who completed the study

*Data* Median (minimum–maximum)

*BDI* Beck Depression Inventory; *HADS* hospital anxiety and depression scale

**Fig. 2:**
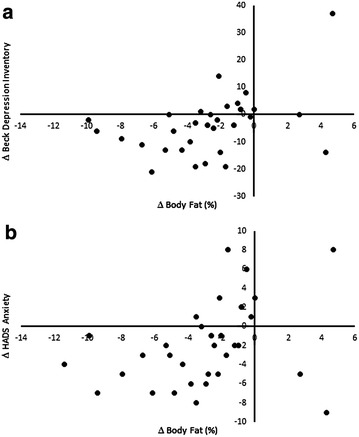
Correlation between body fat and depressive (**a**) and anxiety symptoms (**b**) after IGB treatment. *IGB* Intragastric balloon; *BDI* Beck Depression Inventory; *HADS* hospital anxiety and depression scale; *HADS-A* hospital anxiety and depression scale-anxiety

We used correlation analysis to investigate whether weight loss and modifications in body composition influenced the improvements in psychiatric symptoms and present the results in Table 2. The absolute decrease in the percentage of body fat correlated with improvements in BDI scores (p = 0.008; Fig. 2a) and anxiety symptoms (p = 0.014; Fig. 2b). Interestingly, waist loss correlated significantly with the improvement in anxiety symptoms (p = 0.017), with weight and BMI showing a trend toward significance (p = 0.08 for both).

**Table 2:** Correlation between variations in body composition and psychiatric symptoms after intragastric balloon (IGB) treatment (∆ for all variables)

	BMI (kg/m^2^)	Weight (kg)	Waist (cm)	Total fat (g)	Total fat (%)	Lean mass (g)
BDI	r = 0.11 *p* = 0.48	r = 0.12 *p* = 0.46	r = 0.24 *p* = 0.14	r = 0.20 *p* = 0.26	r = 0.46 *p* = 0.008	r = −0.04 *p* = 0.81
HADS-depression	r = 0.02 *p* = 0.87	r = 0.05 *p* = 0.75	r = 0.23 *p* = 0.16	r = −0.02 *p* = 0.89	r = 0.12 *p* = 0.50	r = 0.05 *p* = 0.77
HADS-anxiety	r = 0.27 *p* = 0.084	r = 0.28 *p* = 0.08	r = 0.38 *p* = 0.017	r = 0.21 *p* = 0.22	r = 0.42 *p* = 0.014	r = 0.22 *p* = 0.21

*DXA* dual-energy X-ray absorptiometry; *BMI* body mass index; *BDI* Beck Depression Inventory; *HADS* hospital anxiety and depression scale; *HADS*-*D* hospital anxiety and depression scale-depression; *HADS*-*A* hospital anxiety and depression scale-anxiety

## Discussion

Several studies have reported a positive impact of weight loss on mental disorders such as depression and anxiety [16–24]. However, the most used parameter of weight loss in these analyses was the BMI, which does not reflect the individual’s body composition. Among accurate methods to measure body composition, DXA has proven to be one of the most reliable in clinical practice due to its high reproducibility, moderate cost, minimal irradiation, and fast execution [31, 32].

To the best of our knowledge, this is the first study to correlate psychiatric symptoms with weight loss and changes in body composition assessed by DXA after 6 months of IGB treatment. In addition to the weight loss already expected with IGB, significant improvements in depressive and anxiety symptoms were evidenced in this specific population. Two additional relevant facts were observed using DXA. First, there was no correlation between decreases in weight, BMI, and WC with improvement in depressive symptoms. However, the decrease in the percentage of total fat correlated with the decrease in BDI, but not with changes in the HADS-depression score. Second, improvements in anxiety symptoms correlated with improvements in both WC and percentage of total fat.

Different treatment options for obesity, such as behavioral and pharmacological therapies, surgery, and IGB have been associated with improvements in mood after weight loss [16–24]. Psychological and interpersonal changes, such as improvements in negative self-esteem, drive for thinness, body dissatisfaction, anxiety, eating disorder, personality disorders (borderline, avoidant, passive-aggressiveness) have been speculated to be directly related to weight loss [23, 33]. It is worth noticing that significant improvements in depressive symptoms have already been observed even with dietetic interventions. In 2008, Kiortsis et al. demonstrated significant improvements in the Hamilton Depression Rating Scale (HAM-D) scores with three different interventions: low-caloric diet (LCD), sibutramine + LCD, and orlistat + LCD [34]. In 2009, Faulconbridge et al. demonstrated that four different interventions for weight loss (three groups receiving sibutramine and one group receiving lifestyle modification alone) led to significant improvements in BDI scores [19]. In 2013, Grilo et al. evaluated the effects of two different interventions (orlistat + a behavioral weight loss [BWL] program versus placebo + BWL) in obese individuals with and without binge eating disorder [35]. Although patients treated with orlistat presented increased weight loss, similar improvements in depressive symptoms were observed in all groups [35]. These studies demonstrate that even mild weight loss, regardless of the implemented intervention, is associated with significant improvements in depressive symptoms. On the other hand, most studies have reported a tendency for a decrease and normalization in psychopathological symptoms following bariatric surgery [36–40]. However, most of these studies have failed to investigate whether these improvements were independently associated with the degree of weight loss.

The IGB is a therapeutic option for the treatment of obesity. In randomized, sham-controlled trials, treatment with liquid-filled IGB promoted weight loss through different mechanisms, including decreased hunger, increased satiety, and modification in eating habits (as a self-educational tool) [40–42]. Short-term studies have reported weight losses between 12 and 15.2 kg after 6 months of IGB, and our results are consistent with these data [39–45]. A few studies have analyzed the body composition after 6 months of IGB using bioelectrical impedance, showing a significant reduction in fat mass and fat-free mass [24, 46, 47]. Bužga et al. used DXA to verify the body composition after treatment with IGB and demonstrated sharp decreases in fat and lean mass [48]. Our patients also presented similar changes in body composition verified by DXA.

As far as we are aware, only one study has investigated changes in depressive symptoms after treatment with IGB [24]. The authors demonstrated that the improvement in depressive symptoms was closely associated with weight loss. However, when depressed patients were divided according to BMI (≤40 or >40 kg/m^2^), neither a difference in depression score nor in depression severity was noted, despite the important difference in body weight and, thus, in body image and self-esteem [24]. Our patients showed a reduction in BDI scores after 6 months with the IGB. They also presented a significant decrease in all anthropometric measures, so it is possible that improvements in self-esteem, perception of corporal image, and life satisfaction could have been reflected in these patients’ depression symptoms. However, the inclusion in the BDI of somatic symptoms to evaluate depression and well-being attributed to weight loss may have influenced this result, as improvements in these symptoms could alleviate physical problems related to obesity. This fact could explain the relationship between the improvement in BDI and the decrease in body fat, but does not explain its lack of association with anthropometric variables.

In addition to our findings regarding BDI and body composition, we were unable to demonstrate an association between the amount of weight loss and the improvement in symptoms of depression with the HADS-depression scale. Somatization could partially explain this difference; obesity may be considered as probably influencing the development and maintenance of somatization disorder (i.e., expressions of physical complaints with no corresponding organic injuries) [8, 49]. The HADS has been developed to avoid interference from somatic disorders on the scale, so anxiety and depression symptoms related with physical diseases were excluded [50]. Taken together the results from the HADS-depression and BDI, it seems that the improvement in depressive symptoms was not directly related to the amount of weight loss, as it occurred in patients receiving an effective treatment for obesity, regardless of the degree of weight loss.

The decrease in anxiety symptoms assessed with the HADS-anxiety after IGB was another finding of our study. Unlike the depressive symptoms, the anxiety symptoms were directly associated with the decrease in WC (with a trend toward significance observed with weight and BMI), as well as in total fat mass. One reasonable explanation is that the improvement in anxiety symptoms is more related to the weight loss per se than the improvement in depressive symptoms, and is independent of changes in total fat, visceral fat, or lean mass.

The present study has some limitations. First, we did not include a control group and our sample comprised a small number of patients. Second, the only inclusion criteria used to define obesity was BMI ≥30 kg/m^2^. Therefore, a very heterogenous sample was selected for this study, with BMI ranging from 30.9 to 53.7 kg/m^2^. Third, only 78% of the sample completed the study (39 individuals). Eleven patients failed to complete the study due to gastric intolerance in four, balloon rupture in five, uterus cancer in one, and loss to follow-up in another. It is worth noticing that balloon rupture, a rare complication of the procedure, occurred in 10% of the sample. Finally, the significant number of patients who were unable to tolerate the device (8%) indicates that significant gastric intolerance may be one of the most important adverse events related to the IGB. In contrast, our study had important strengths, including the use of DXA to analyze the body composition and the adoption of two different questionnaires to evaluate the occurrence of mood disorders.

## Conclusions

In conclusion, a 6-month treatment of individuals with obesity and MS using IGB was associated with significant weight loss and significant improvements in depressive and anxiety symptoms. By using DXA, we also demonstrated that the improvement in psychiatric symptoms was associated with the decrease in the percentage of body fat, but not with the anthropometric parameters, including BMI and WC. Further studies are necessary to clarify the role of body fat and weight loss in mental health.

## Abbreviations

BMI: body mass index
BDI: Beck Depression Inventory
BWL: behavioral weight loss
DXA: dual-energy X-ray absorptiometry
HADS: hospital anxiety and depression scale
HAM-D: Hamilton depression rating scale
IGB: intragastric balloon
MS: metabolic syndrome
LCD: low-caloric diet
WC: waist circumference
